# YouTube as a Source of Information in Trauma Management for ATLS (10th Edition) Guidelines: Evaluation of Trauma Management Videos on YouTube

**DOI:** 10.1155/2024/7077469

**Published:** 2024-10-24

**Authors:** Merve Yazla, Seyma Handan Akyon, Esin Aslı Aybayar, Seyda Gedikaslan, Lukasz Szarpak, Omer Faruk Turan, Jacek Smereka, Mustafa Ekici, Abdullah Osman Kocak, Burak Katipoglu

**Affiliations:** ^1^Ankara Etlik City Hospital, Clinic of Emergency Medicine, Ankara, Turkey; ^2^Bilkent Gölpazarı State Hospital, Family Medicine, Bilecik, Turkey; ^3^Henry JN Taub Department of Emergency Medicine, Baylor College of Medicine, Houston, Texas 77030, USA; ^4^Department of Emergency Medical Service, Wroclaw Medical University, Wroclaw 50-367, Poland; ^5^Health Services, Aydın, Turkey; ^6^Balıkesir Atatürk City Hospital, Clinic of Emergency Medicine, Balıkesir, Turkey

**Keywords:** assessment of trauma, ATLS, management of trauma, trauma, video, YouTube

## Abstract

**Background:** Trauma is one of the leading causes of mortality worldwide, and online platforms have become essential sources of information for trauma management. YouTube can play a significant role in helping people access medical information.

**Methods:** YouTube was searched using the keywords management of trauma and assessment of trauma to identify relevant videos. Two authors independently evaluated the videos according to the ATLS (10th edition) guidelines, the modified DISCERN (m-DISCERN) scale, and the Global Quality Scale (GQS) criteria. The videos that met the study criteria were evaluated based on the provider, video length, and view count.

**Results:** Out of 939 videos, 667 were excluded resulting in 272 videos included in the study. According to the ATLS (10th edition) guidelines, the median score for videos was 8 (IQR 7-8). Videos uploaded by official institutions and healthcare professionals received higher scores than from uncertain sources (*p* = 0.003). According to the GQS, 86% of the videos were low or moderate quality; uncertain sources uploaded 78% of low-quality videos.

**Conclusion:** YouTube is an information source about trauma management that contains videos of varying quality and has a broad audience. Official institutions and healthcare professionals should be aware of this evolving technology and publish up-to-date, accurate content to increase awareness about trauma management and help patients distinguish helpful information from misleading content.

## 1. Introduction

Trauma-related mortality is a significant public health issue [1]. In different settings including developing countries, the health status of many patients, particularly in rural areas, can critically deteriorate before reaching emergency medical services due to longer transfer times; therefore, rapid and accurate first-aid intervention in trauma patients at the scene can be life-saving [2–6].

It has been suggested that some trauma-related deaths could be prevented with basic first-aid procedures for trauma patients [7, 8]. Various studies have demonstrated that early and adequate cardiopulmonary resuscitation (CPR) by lay rescuers in witnessed nontraumatic cardiac arrest cases improving outcomes in prehospital cardiac arrest patients [9, 10]. Rapid and accurate intervention by lay rescuers and healthcare professionals can decrease mortality and morbidity in trauma patients by ensuring safety at the accident scene, maintaining cervical stabilization, ensuring airway patency, performing CPR, controlling external bleeding, and preventing hypothermia [11].

Potential lay rescuers may have insufficient knowledge to respond to urgent health problems that occur in their surroundings, and as a result, they can seek medical information from alternative sources [12]. The Internet has emerged as the primary source for obtaining such information. The World Wide Web provides broad information for patients and their relatives to access health-related information. More than half of patients use the Internet to get medical information [13]. Web-based platforms that facilitate content and information sharing have revolutionized global information sharing [14]. This significant transformation has led to numerous innovations in accessing, disseminating, and conducting information-related educational activities. Even beyond patients and the general population, medical students and healthcare professionals use the Internet and social media to get medical information [15].

YouTube, the largest video search engine on the Internet, is primarily used for sharing videos including social content, and the platform also hosts visual content for educational purposes [16, 17]. Despite the existing challenges, the readily available and expeditious accessibility of the Internet provides a promising potential for playing a crucial role in facilitating individuals' acquisition of medical knowledge. However, these visual materials can be published directly without undergoing any control mechanism. As a result, individuals may not be aware of the accuracy and reliability of this information, and they might need to grasp the significance of this issue fully [18].

The aim of this study was to investigate the reliability, quality, and information content of ATLS scenarios related to trauma management on YouTube.

## 2. Materials and Methods

The study was approved by the Ethics Committee of the XXX City Hospital (E-30041352-514.19.99-213995999) and conducted by the guidelines of the Declaration of Helsinki.

This study had a cross-sectional analytical design. English videos uploaded to YouTube from January 1, 2018, to April 1, 2023, containing the keywords “management of trauma” and “assessment of trauma” based on the ATLS (10th edition) publication date, were searched. After applying the exclusion criteria, the remaining videos were reviewed.

The exclusion criteria included nonmedical content (advertisements, news, and interviews), videos in languages other than English, videos containing advertisements, live-action footage lacking educational content, comedy or entertaining content unrelated to educational purposes, videos consisting of interviews or news content, duplicate videos (videos uploaded by different users but containing the same content or shorter versions of the same video), videos not involving trauma scenarios, videos covering nontrauma topics, and videos uploaded before January 1, 2018.

The videos that met the study criteria were evaluated based on the uploader, video length, and view count. All videos included in the study were independently reviewed by two authors (emergency physicians). In cases of disagreement between the two emergency physicians, a third specialist was consulted.

The videos were evaluated using the ATLS (10th edition) guidelines, the modified DISCERN (m-DISCERN), and the Global Quality Scale (GQS) criteria.

Initially, the physicians assessed the videos according to 10 criteria based on the ATLS (10th edition) guidelines and awarded 1 point for each criterion covered by the video (Table 1). A score of eight or higher could be considered a sufficient educational video rating.

The quality and reliability of the videos included in the study data were evaluated using the “GQS” and the “m-DISCERN” scales, utilized in several internet-based studies before [19–22].

The DISCERN scale was designed to assess the quality of written information about treatment options for any health condition used by individuals seeking healthcare services and information providers. The m-DISCERN scale (m-DISCERN) consisting of five questions was utilized to evaluate visual media and visual information. Each question received a score of 1 for “yes” answers and 0 for “no” answers resulting in a scale where the highest score is five and the lowest is 0. This scoring system evaluated the video's objectivity, reliability, and comprehensibility from the perspective of information sources [23, 24].

The reliability and integrity of the information presented in the content were assessed using the m-DISCERN scale. Each question received a score of 1 for “yes” and 0 for “no” (Table 2).

The GQS is a scoring system developed by Bernard et al. to assess the quality of information obtained from Internet sources. It measures the quality of the video content. The scale ranges from one to five, with five indicating the highest quality, representing videos with clear and comprehensive information, while one shows very low quality, with most information missing [20].

The GQS was used to evaluate the video quality. The scoring ranges from 1 to 5, where 1-2 points indicate low quality, 3 points indicate moderate quality, and 4-5 points indicate high video quality. The scoring definitions are shown in Table 3.

### 2.1. Statistical Analysis

SPSS 23.0 statistical software package (IBM, Armonk, NY, USA) was used to analyze the research data. Continuous variables were expressed as mean ± standard deviation (SD), while categorical variables were presented as numbers and percentages. The normality assumption for numerical variables was evaluated both analytically (the Kolmogorov–Smirnov test and the Shapiro–Wilk test) and graphically (histogram). Since the data did not follow a normal distribution, The Mann–Whitney *U* test was used for the binary group comparison of numerical variables, and the Kruskal–Wallis test was used for comparisons involving three or more groups. *p* values less than 0.05 were considered statistically significant.

## 3. Results

**Figure 1:**
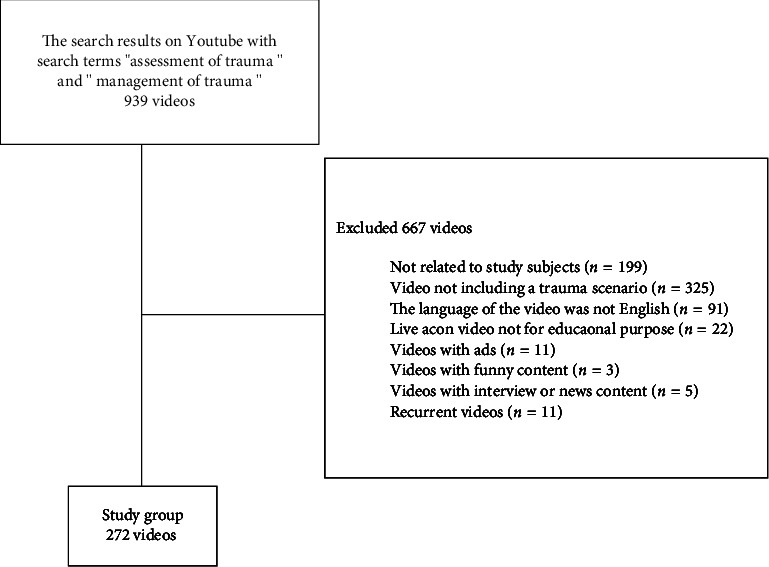
Video flowchart.

Of the 939 videos obtained from the search using specific keywords, 667 were excluded as they did not meet the study criteria. The remaining 272 videos formed the study group (Figure 1) and were watched and evaluated by two independent emergency physicians. In videos that were scored differently by two evaluators, the final decision was made by a third person.

One million two hundred seventy-one thousand eight hundred thirteen views were recorded for the 272 eligible videos in the study. The median number of views or clicks is 125 (with an interquartile range (IQR) of 62–396). The median length of the videos was 419 s (IQR 305–567). The characteristics of the videos included in the study are presented in Table 4.

The videos' median score was 8 (with an IQR of 7-8) according to the compliance of the ATLS (10th edition) guidelines. The comparison of video scores based on different variables is presented in Table 5. The scores were significantly different between groups (*p* < 0.001). In binary comparisons, videos uploaded by official institutions and healthcare professionals had higher scores compared to the unclassified group (*p*=0.003). There was no significant difference in scores between videos uploaded by official institutions and healthcare professionals (8 [IQR 8-9] and 8 [IQR 7-9], *p*=0.963).

The median number of views for videos uploaded by official institutions was 16,109 (with an IQR of 1610-110,291), for healthcare professionals was 238 (with an IQR of 88-3155), and for unclassified sources was 108 (with an IQR of 56–184). In the analysis that assesses the number of views based on the video sources, statistically significant differences were found in all three groups (*p* < 0.05).

Out of the 76 videos containing misinformation or incorrect practices (GQS = 1 and 2), 60 were uploaded by unidentified individuals, and healthcare professionals uploaded 16 on YouTube. These videos had lower view counts compared to the other videos, and a statistically significant difference was found in view counts when compared to the view counts of other videos (114 [IQR 58-235] and 1286 [IQR 136-11889], *p* < 0.05).

According to the GQS scale, 86% of the videos were of low or moderate quality. When comparing the sources that uploaded the videos, a statistically significant difference was found (*p* < 0.05). Only 3.3% of the videos uploaded by unidentified sources were of high quality, while 85.7% of the videos uploaded by official institutions were of high quality. In addition, there were no low-quality videos among those uploaded by official institutions. Unidentified sources uploaded 78% of the low-quality videos. Among the videos uploaded by unidentified sources, only 3% were of high quality.

When examining the scales used to assess the quality of videos, it was found that only five videos provided additional information sources for users according to the m-DISCERN scale, and seven videos addressed controversial or uncertain topics. When comparing the sources that uploaded the videos on YouTube, a significant difference was found in the scores according to the m-DISCERN scale. The average scores of the m-DISCERN scale were significantly higher in videos uploaded by healthcare professionals than those uploaded by unidentified individuals (*p* < 0.05).

## 4. Discussion

YouTube is a popular video-sharing platform where uploading and accessing videos is fast, free, and easy. Patients and healthcare professionals use video-sharing sites such as the Internet and YouTube at increasing rates and frequency to obtain information on health-related issues [25]. Although platforms such as YouTube are seen as potential sources of information, it is sometimes difficult to find the content we seek from the right sources. In our study, 71% of the videos did not meet the inclusion criteria. In previous studies involving the reliability of YouTube videos, 80%–94% of the videos were excluded [15, 26–29]. Although this rate has decreased slightly in our study, 3 out of 4 videos still need to show the content we seek. The high exclusion rate makes it difficult to find the necessary content on a video-sharing site such as YouTube, which has reached many views. In addition, this problem is partially solved by developing a particular infrastructure according to the educational platforms of sharing sites such as YouTube and adding the options to determine the content according to the researched subject.

Trauma is one of the leading causes of mortality worldwide. Online platforms have also become a source of information on trauma management. One of the online platforms, YouTube, has the potential to play a significant role in facilitating individuals' access to information about medical conditions. In this study, we analyzed the content and quality of the information in the trauma management videos on YouTube according to the ATLS (10. Ed) guidelines. Studies on visual scientific content on the Internet are evaluated with scales such as m-DISCERN. The information's currency, accuracy, and quality are determined by the scores formed after the evaluation. GQS, on the other hand, is a five-point Likert scale based on the quality of information, the flow of information presented online, and ease of use [20–24, 30, 31]. Therefore, GQS and m-DISCERN scales were used in our study. Our study, which we examined according to these two scales and the ATLS (10th edition) guideline, determined that YouTube videos were insufficient in providing consistency and video quality according to current information and guidelines on trauma management.  Table 6 presents the key studies that summarize the scientific evidence cited in discussing our results.

In the study of Şahin et al., independent users uploaded the vast majority of videos containing low-quality medical information. It has been determined that the videos uploaded by health professionals to YouTube are of higher quality than those uploaded by independent individuals, and they have received higher scores according to the scales [32]. According to the GQS scale, 86% of the videos examined in our study have low and medium quality. Unknown users uploaded 76% of low- and medium-quality videos. As a result of the rapid introduction of digital applications into our lives, free-of-charge platforms such as YouTube that can reach large audiences have become a source of medical information [33]. Therefore, low-quality videos containing false information uploaded by uncertain individuals endanger public health. To prevent this negative situation, videos with high scores from the GQS and m-DISCERN scales uploaded by health professionals and official institutions should be prioritized and kept in the foreground in YouTube's software algorithm.

In a study by Sing et al. to characterize the content and quality of information about rheumatoid arthritis on YouTube, it was found that nonprofit and academic videos were the best sources of information. However, these videos only represent 12.7% of the total videos. In addition, it was found that the viewing rate of these videos was only 13.4% [24]. There are also studies showing that most of those who watch health-related videos on the Internet are interested in something other than the source of the video. This also implies that people receive health advice without consistently examining the quality indicators of the information they find online [34]. Considering that unknown users uploaded 67% of the videos examined in our study, health-related guiding and informative videos uploaded by nonexperts in the field should be monitored and restricted by YouTube. Videos of such low quality and misleading content should only be permitted for publication after undergoing thorough review and evaluation.

None of the videos uploaded by official institutions scored below eight according to the ATLS (10th edition) guidelines. In addition, according to the GQS scale, it was determined that the content uploaded by official institutions had no low-quality videos. According to the m-DISCERN scale, videos uploaded by healthcare professionals and unidentified people had the lowest score of 0, although the videos of official institutions did not score below 2 points. This shows that the videos of official institutions are much more qualified, and accurate information can be accessed. However, the videos uploaded by official institutions constitute only 2% of all videos. To change this situation, it is crucial to create more videos adhering to standardization guidelines provided by relevant official institutions. These videos should be informative, concise, easily understandable, unbiased, and address contentious topics while offering essential supplementary resources.

In the survey studies, it was found that 86% of the Internet users accessing health-related information believe that the information they have obtained is reliable, and 64% stated that this information affects the decision they will make about the treatment [35]. The medical information obtained over the Internet is increasing exponentially every day, and it is seen that it affects patients' decisions. In addition, since there is no control mechanism, this information can be provided to the Internet by many different sources, as seen in our study. This situation leads to questioning the reliability, adequacy, and quality of medical information obtained online [36]. The reliability of medical information is still unknown. In our study, 56% of the videos received a score of 8 or higher according to the ATLS (10th edition) guidelines, yet 86% were rated as low or moderate quality based on the GQS. In addition, only 1.1% of the videos obtained the highest scores of 4-5 points according to the m-DISCERN scale. For this reason, there is a need for up-to-date, reliable, sufficient, and quality videos.

Although the overall image/broadcast quality of YouTube videos is better with the developing technology, there are videos with insufficient content according to our study's ATLS (10th edition) guidelines. Therefore, scoring on the GQS scale, where video quality and content are evaluated together, caused low scores due to lack of content. Also, in the m-DISCERN scale, not specifying additional information sources for viewers and not questioning ambiguous and controversial issues cause high-quality and very useful videos to receive low scores. The medium-scale videos watched by emergency medicine physicians specializing in trauma do not mean that these videos are insufficient for the public. Videos included in the study are unsuitable for healthcare professionals' use as they must meet the academic and scientific criteria set by GQS, m-DISCERN, and the latest guidelines. The rationale is that videos scoring 4-5 points according to the m-DISCERN scale constitute only 1.1% of the videos. In popular social media channels such as YouTube, content producers create concise and summarized videos to attract a larger audience. Videos are crafted to be captivating, concise, and aligned with current guidelines due to concern for higher viewership. They avoid controversial subjects and content that redirect viewers to supplementary resources. This causes videos to receive low scores on the m-DISCERN scale.

More than half of Internet users say the information they find influences their decisions about how to treat a disease or condition. On the other hand, 35% of people say that the information they reach affects their decision to see a physician [34]. According to the GQS scale, 86% of the videos examined in our study are of low to medium quality. Unknown individuals uploaded 67.6%, and 27.9% contained false information. Therefore, to increase the online accessibility of accurate medical information, government agencies and health professionals should emphasize that people should be selective when accessing medical information from the web. Government agencies and healthcare professionals should provide basic guidance on content evaluation, such as assessing information based on the Medical Library Association's user's guide to finding and evaluating health information on the web [37].

By the literature, the results of our study also showed that the videos uploaded by official institutions and health professionals contain more accurate information [26, 38–40]. The median score of videos uploaded by official institutions and healthcare professionals was found to be 8, while the median score of videos uploaded by unknown individuals was 7. In addition, videos uploaded by official institutions and health professionals were watched 150 times more than videos uploaded by unknown people. This suggested that the videos uploaded by unknown individuals were less watched as they did not inspire confidence.

No significant relationship was found between the number of video views and video scores. Similarly, no relationship was found between the number of video views and video quality in studies conducted on different subjects [38–40]. However, a statistically significant difference was found between the video source and the number of views in all three groups (*p* < 0.05). While the number of views of official institutions was the highest, the number of views of unknown individuals was the lowest. In addition, the videos uploaded by unknown individuals do not have more than 10,000 views. This indicates that users tend to disregard health advice from unreliable sources and place more trust in content provided by official institutions. Consequently, videos from official institutions are more reliable.

Our study has several limitations. First, our study was limited to a single snapshot analysis of English-only videos on YouTube. Only the videos during the study period were examined. In addition, the fact that other channels and websites that provide health information were not examined can be considered a limitation of our study. In addition, it would be possible to find trauma-related educational videos on various channels using keywords other than the determined ones.

In other studies in the literature, video evaluations were made with criteria such as the first three pages, the first ten pages, or the first 200 videos [22, 24, 41, 42]. This limitation was removed in our study, and all videos from ATLS (10th edition) published from 2018 to the present day were evaluated.

## 5. Conclusion

Healthcare professionals and individuals should not consider YouTube videos on trauma management as a completely reliable source of information. Official institutions and healthcare professionals should proactively contribute to disseminating reliable information on trauma management and preventing misinformation by publishing updated videos from reliable and scientific sources, based on current guidelines.

## Figures and Tables

**Table 1:** Scoring criteria according to ATLS (10th edition) guidelines.

**Intervention**	**Score**
Ensuring the safety of the area where the trauma occurred	1
Triage and preparation	1
A (airway)	1
B (breathing)	1
C (circulation)	1
D (disability)	1
E (exposure)	1
Resuscitation	1
Patient transfer	1
Secondary evaluation	1

**Table 2:** Scoring criteria according to the m-DISCERN scale.

**Definition**	**Score**
Are the aims clear and achieved?	1
Are reliable sources of information used?	1
Is the information presented both balanced and unbiased?	1
Are additional sources of information listed for patient reference?	1
Are areas of uncertainty mentioned?	1

**Table 3:** Global Quality Scale.

**Definition**	**Score**
Poor quality, poor flow of the site, most information missing, and not at all useful for patients	1
Generally poor quality, poor flow, some information listed but many important topics missing, and of very limited use to patients	2
Moderate quality, suboptimal flow, some important information is adequately discussed but others poorly discussed, and somewhat useful for patients	3
Good quality and generally good flow; most of the relevant information is listed, but some topics are not covered, useful for patients	4
Excellent quality and excellent flow and very useful for patients	5

**Table 4:** Characteristics of the videos included in the analysis.

**Date (year)**	***N***	**%**
2018	29	10.7
2019	40	14.7
2020	63	23.2
2021	57	21.0
2022	72	26.5
2023	11	4.0
Video source		
Official institution	7	2.6
Healthcare professionals (physicians and medical staff)	81	29.8
Unidentified (unclassified)	184	67.6
Number of views		
<10.000	258	94.9
≥10.000	14	5.1
Contains misinformation or incorrect practices		
No	196	72.1
Yes	76	27.9
The total scores obtained according to ATLS		
<5	26	9.6
5–7	92	33.8
≥8	154	56.6

**Table 5:** Comparison of video sources according to variables.

**Variables**	**Video source**		
	**Official institution**	**Healthcare professional**	**Unidentified**
Number of views (median-IQR)	16,109 (1610–110291)	238 (88.5–3115)	108 (56–184)
Video duration (median-IQR)	566 (476–1279)	507 (370–776)	384 (286–520)
GQS *n* (%)			
Median score (IQR)	8 (8–9)	8 (7–9)	7 (6–8)
Low quality	0 (%0)	16 (%19.8)	60 (%32.6)
Moderate quality	1 (%14.3)	39 (%48.1)	118 (%64.1)
High quality	6 (%85.7)	26 (%32.1)	6 (%3.3)
m-DISCERN *n* (%)			
0	1 (%14.3)	1 (%1.2)	4 (%2.2)
1	0	13 (%16)	17 (%9.2)
2	6 (%85.7)	64 (%79)	143 (%77.7)
3	0	2 (%2.5)	18 (%9.8)
4	0	0	2 (%1.1)
5	0	1 (%1.2)	0
ATLS			
<5	1 (%14.3)	9 (%11.1)	16 (%8.7)
5–7	3 (%42.9)	35 (%43.2)	54 (%29.3)
≥8	3 (42.9)	37 (%45.7)	114 (%62)

**Table 6:** Key studies on YouTube videos as a source of medical information.

	**Published year**	**Search term**	**Included videos/the number of videos**	**Type of the uploader/sources of videos**	**Evaluation of video parameters/analysis of content**
YouTube for information on rheumatoid arthritis—a wakeup call	2012	Rheumatoid arthritis	April 8, 2011 The first 200 videos (first 10 pages) 102 YouTube videos	Independent users Government/news agencies University channels/professional organizations health information websites Medical advertisements/for-profit companies	GQS DISCERN
Evaluation of the educational value of YouTube videos about physical examination of the cardiovascular and respiratory systems	2013	Cardiovascular system examination Cardiovascular clinical examination Cardiovascular physical examination Respiratory system examination Respiratory clinical examination Respiratory physical examination	December 2, 2011 The first 10 pages of results for each search term 56 YouTube videos	—	The textbook and video by Talley and O'Connor, “clinical examination” scientific content Technical aspects Authority/creator pedagogy used
YouTube as an educational tool regarding male urethral catheterization	2014	Male urethral catheterization urinary catheter insertion Male catheter insertion	The first 5 pages (first 100 videos) 49 YouTube videos	Patient experience Surgical technique Physician news report	Safe catheter insertion score
Are YouTube videos accurate and reliable on basic life support (BLS) and CPR	2014	CPR BLS	2011–2013, 209 YouTube videos	Private agency Guideline bodies such as AHA/Red Cross/ERC Individual identifying him/herself as an emergency medical technician, certified CPR instructor, or physician Individual with credentials unspecified news programme	2010 CPR guidelines
YouTube as a potential training method for laparoscopic cholecystectomy (LC)	2015	Laparoscopic cholecystectomy	March 1, 2014 The first 100 videos 73 YouTube videos	Primary center Secondary center Tertiary center and academic institution Commercial institution	LC guidelines An arbitrary score system for video quality, devised from existing LC guidelines
Evaluation of educational content of YouTube videos relating to the neurogenic bladder and intermittent catheterization	2015	Neurogenic bladder intermittent catheter Spinal cord injury intermittent catheter	June 29, 2014 The first 50 results for each search term (50 × 2 = 100 videos) 71 YouTube videos	Healthcare provider Health advocacy group Patient Merchant News agency Uncertain	European Urological Association guidelines and established clinical practice Video marketing characteristics
Reliability of trauma management videos on YouTube and their compliance with ATLS (9th edition) guidelines	2018	Assessment of trauma Management of trauma	January 2011–June 2016 67 YouTube videos	Institutional (university and society) Individuals other than healthcare personnel Healthcare personnel (doctors and paramedics) Unknown (not classified)	ATLS (9th edition) guidelines
Assessment of the accuracy of cardiopulmonary resuscitation videos in English on YouTube according to the 2015 AHA resuscitation guidelines	2019	CPR Cardiopulmonary resuscitation basic life support	15 October 2015–21 October 2016 92 YouTube videos	Medical organizations Healthcare professionals or organizations Unidentified sources	2015 AHA resuscitation guidelines
YouTube as a source of information in retinopathy of prematurity	2019	Retinopathy of prematurity ROP	Between April 14 and 25, 2018 The first ten pages The first 100 relevant Videos 100 YouTube videos	Surgeon/practitioner Independent user Hospital/free clinic Social media/TV Medical site University Advertisement	Any aspect of ROP
YouTube as a source of information on COVID-19 and rheumatic disease link	2020	COVID-19 Rheumatology SARS COVID-2 Rheumatology Coronavirus 2019 Rheumatology COVID-19 Arthritis SARS COVID-2 arthritis Coronavirus 2019 arthritis	2 April 2020 The first three pages for each search term (60 × 6 = 360 videos) 46 YouTube videos	Society/non-profit organization Physician Health-related website University/academic Patient/independent User Nonphysician healthcare personnel Pharmaceutical company News agency	GQS mDISCERN The like ratio
Evaluation of the reliability and quality of YouTube videos as a source of information for transcutaneous electrical nerve stimulation	2023	Transcutaneous electrical nerve stimulation	The first 100 videos on October 30 2022, 100 YouTube videos	Academic sources Society/professional organizations Physicians Commercial and health-related websites	JAMA mDISCERN GQS VPI
Assessment of quality and reliability of YouTube videos for patient and physician education on inflammatory myositis	2023	Myositis Idiopathic inflammatory Myositis Dermatomyositis Polymyositis Cancer-associated myositis Inclusion body myositis Immune-mediated necrotizing Myopathy Juvenile dermatomyositis Overlap idiopathic inflammatory myositis	March 2021 The first 100 videos for each term 453 YouTube videos	Hospital Group practice or physician Nonmedical independent user Nonmedical media organization Professional medical body/patient support group Pharmaceutical company	mDISCERN GQS JAMA
An evaluation of male rhinoplasty videos on YouTube and TikTok: A DISCERN analysis	2024	Male rhinoplasty Male nose job	264 YouTube videos 165 TikTok videos	Patient Physician Healthcare group Academic institution/society Entertainment	DISCERN

Abbreviations: AHA, American Heart Association; ATLS, advanced trauma life support; BLS: basic life support; CPR, cardiopulmonary resuscitation; GQS, Global Quality Sale; JAMA, Journal of American Medical Association benchmark criteria; mDISCERN, modified DISCERN scale; ROP, retinopathy of prematurity; VPI, video power index (VPI = [like count/[like count + dislike count] × 100]). The like ratio: like/(like + dislike).

## Data Availability

The datasets used and/or analyzed during the current study are available from the corresponding author on reasonable request.
